# Supplementary material for: Traditional Gender Role Attitudes and Job-Hunting in Relation to Well-Being: A Cross-Sectional Study of Japanese Women in Emerging Adulthood
Source: Int J Environ Res Public Health. 2025 Sep 4;22(9):1385. doi: 10.3390/ijerph22091385 (PMC12469634; doi:10.3390/ijerph22091385)
Supplement: Supplementary file 1 [file ijerph-22-01385-s001.zip › ijerph-3759267-supplementary.pdf]

Table S1. Correlation between well-being and variables (N = 132).

**Variable**			
Job hunting		0.05	
(*p*-value)	(	0.58	)
Gender-role attitudes		0.05	
(*p*-value)	(	0.54	)
Age		0.03	
(*p*-value)	(	0.78	)
Occupation		0.11	
(*p*-value)	(	0.20	)
Household situation		−0.01	
(*p*-value)	(	0.91	)
Present illness		0.20	
(*p*-value)	(	0.02	)
Self-assessed living conditions		−0.19	
(*p*-value)	(	0.03	)
Personality (Extraversion)		0.31	
(*p*-value)	(	0.00	)
Personality (Agreableness)		0.23	
(*p*-value)	(	0.01	)
Personality (Conscientiousness)		0.12	
(*p*-value)	(	0.17	)
Personality (Neuroticism)		−0.38	
(*p*-value)	(	0.00	)
Personality (Perceived social support)		0.11	
(*p*-value)	(	0.22	)
Perceived social support		0.57	
(*p*-value)	(	0.00	)
